# An Alternative to the Light Touch Digital Health Remote Study: The Stress and Recovery in Frontline COVID-19 Health Care Workers Study

**DOI:** 10.2196/32165

**Published:** 2021-12-10

**Authors:** Sarah M Goodday, Emma Karlin, Alexandria Alfarano, Alexa Brooks, Carol Chapman, Rachelle Desille, Shazia Rangwala, Daniel R Karlin, Hoora Emami, Nancy Fugate Woods, Adrien Boch, Luca Foschini, Mackenzie Wildman, Francesca Cormack, Nick Taptiklis, Abhishek Pratap, Marzyeh Ghassemi, Anna Goldenberg, Sujay Nagaraj, Elaine Walsh, Stress And Recovery Participants, Stephen Friend

**Affiliations:** 1 4YouandMe Seattle, WA United States; 2 Department of Psychiatry University of Oxford Oxford United Kingdom; 3 Nationwide Children's Hospital Columbus, OH United States; 4 MindMed New York, NY United States; 5 Tufts University School of Medicine Boston, MA United States; 6 Dalla Lana School of Public Health University of Toronto Toronto, ON Canada; 7 School of Nursing University of Washington Seattle, WA United States; 8 Evidation Health Inc San Mateo, CA United States; 9 Cambridge Cognition Cambridge United Kingdom; 10 Department of Psychiatry University of Cambridge Cambridge United Kingdom; 11 Krembil Center for Neuroinformatics Center for Addiction and Mental Health Toronto, ON Canada; 12 Vector Institute Toronto, ON Canada; 13 University of Washington Seattle, WA United States; 14 King's College London London United Kingdom; 15 Institute for Medical Engineering and Science MIT Cambridge, MA United States; 16 Department of Electrical Engineering and Computer Science MIT Cambridge, MA United States; 17 The Hospital for Sick Children Toronto, ON Canada; 18 Department of Computer Science University of Toronto Toronto, ON Canada; 19 Canadian Institute for Advanced Research Toronto, ON Canada

**Keywords:** stress, wearable, digital health, frontline, COVID-19, health care worker, alternative, design, app, assessment, sensor, engagement, support, knowledge

## Abstract

**Background:**

Several app-based studies share similar characteristics of a light touch approach that recruit, enroll, and onboard via a smartphone app and attempt to minimize burden through low-friction active study tasks while emphasizing the collection of passive data with minimal human contact. However, engagement is a common challenge across these studies, reporting low retention and adherence.

**Objective:**

This study aims to describe an alternative to a light touch digital health study that involved a participant-centric design including high friction app-based assessments, semicontinuous passive data from wearable sensors, and a digital engagement strategy centered on providing knowledge and support to participants.

**Methods:**

The Stress and Recovery in Frontline COVID-19 Health Care Workers Study included US frontline health care workers followed between May and November 2020. The study comprised 3 main components: (1) active and passive assessments of stress and symptoms from a smartphone app, (2) objective measured assessments of acute stress from wearable sensors, and (3) a participant codriven engagement strategy that centered on providing knowledge and support to participants. The daily participant time commitment was an average of 10 to 15 minutes. Retention and adherence are described both quantitatively and qualitatively.

**Results:**

A total of 365 participants enrolled and started the study, and 81.0% (n=297) of them completed the study for a total study duration of 4 months. Average wearable sensor use was 90.6% days of total study duration. App-based daily, weekly, and every other week surveys were completed on average 69.18%, 68.37%, and 72.86% of the time, respectively.

**Conclusions:**

This study found evidence for the feasibility and acceptability of a participant-centric digital health study approach that involved building trust with participants and providing support through regular phone check-ins. In addition to high retention and adherence, the collection of large volumes of objective measured data alongside contextual self-reported subjective data was able to be collected, which is often missing from light touch digital health studies.

**Trial Registration:**

ClinicalTrials.gov NCT04713111; https://clinicaltrials.gov/ct2/show/NCT04713111

## Introduction

### Background

The ubiquity of smartphones and the growing availability of wearable sensors has enabled a new era of digital health research [1-3]. The importance of this new form of remote health research has never been more apparent in light of the COVID-19 pandemic, which posed unprecedented challenges for the conduct of *traditional research* involving face-to-face contact [4]. Wearable devices such as smartwatches, smart rings, and bands enable the passive collection of broad semicontinuous physiological and activity information. Smartphones enable the collection of passive information in the form of activity tracking, phone use, and social media patterns, and high-frequency active tasks, surveys, and ecological momentary assessments. The benefits of remote digital health research involving these digital technologies include access to large sample sizes, cost efficiency, elimination of the necessity for travel, and ease of data collection owing to the capabilities to collect passive data. Participants can be remotely enrolled via eConsent frameworks [5] and recruited by social media channels. Accordingly, recruitment, consent, and onboarding can be conducted entirely outside of the clinic or site through the convenience of smartphones. Further, remote digital technologies enable the collection of rich multimodal data involving self-reported subjective and objective measured indicators of disease at semicontinuous or high frequencies. Importantly, these high-resolution data captures are possible outside health care or research visits in real-world settings. Yet, maintaining participant engagement throughout a digital health longitudinal study has proven a challenge [6-8]. The rapport built and *safety net* provided by the in-person visit represent a difficult gap to fill, particularly with additional remote technology usability challenges.

Several large app-based studies are described in the literature [9-11]. These studies share similar characteristics of a *light touch* approach that recruit, enroll, and onboard via a smartphone app and attempt to minimize burden through low-friction active study tasks while emphasizing the collection of passive data. Concerningly, there is strikingly low retention and adherence rates in app-based remote studies. Over half of participants tend to drop out after the first week of participation, while attrition and adherence differs significantly by important sociodemographic factors [6]. In addition to problems with engagement, there are common selection biases across studies tending to enroll White, university/college-educated participants with higher rates of women, reflecting nongeneralizable samples [6]. Further, patients more likely to use digital health trackers are more adherent to chronic disease medication use, suggesting those unlikely to engage in some digital tools may reflect less healthy populations [11]. Predictors of low digital engagement include lack of usability and accessibility, participant privacy and security concerns, perceived utility and motivation, and lack of support [7,8,12]. Although the *light touch* approach minimizes human contact with participants through fully remote enrollment and follow-up via an app, the lack of “human-in-the-loop” and clear value proposition for participants may inadvertently lower their engagement. Digital health cohorts being recruited via a clinic referral compared to recruitment conducted entirely through an app show higher rates of retention and adherence [6]. The *light touch* approach also minimizes the collection of self-reported subjective data to reduce participant burden and in turn attrition, yet this information is crucial to validate objective measured information. This is particularly important given that the field is in an early phase with the need to validate objective measured sensor readouts.

The use of participant incentives and rewards still prevail as one of the most used components for a successful engagement strategy. The use of smartphones makes personalized rewards and reminders possible [13]. Leveraging behavioral psychology, informed strategies for reward scheduling, smartphones can incentivize adherence through rewards for study task completion. Participant tailored push notifications for task reminders can be implemented and have been noted as preferred by participants in digital health research [14]. Beyond incentives, treating participants differently than the traditional blinded *participant* and including them as codrivers in the research process, could prove a powerful way to engage, retain, and accelerate learning for long-term engagement.

The shift to participatory research that tends to involve patient advisory groups who provide input on study design documents such as consent forms [15] and study protocols is already being conducted. However, in the context of digital health and in the use of digital technologies, these participatory models tend to only include users after the relevant technology has been developed. These approaches can be extended further, described by some as “user centered designs,” where participants and patients might be included from the early design to implementation phases and might help shape how the technology is used [16]. Further, participant-centered initiatives [17,18] aim to include participants as equal partners in the entire research process in testing the feasibility of digital technologies for health and wellness. Two recent app-based studies [14] involving a patient- and citizen-centric design, achieved increased retention and adherence when compared to that typically reported in such studies, which demonstrates the promise of these more patient-centered approaches.

Here, we describe an example of an alternative to the *light touch* digital health study that involves active app- and wearable-based assessments coupled with an extensive digital engagement participant-centric strategy. Our objective was to demonstrate that participant-centric engagement approaches might enable a digital health study with improved participant experience, likelihood to retain in the study, and adherence to protocols.

### A Case Example: The Stress and Recovery in Frontline COVID-19 Health Care Workers Study

The COVID-19 pandemic has caused unprecedented stress on health care systems in affected countries and, in particular, on the health care workers working directly with patients with COVID-19. Health care providers faced and continue to face numerous stressors relating to higher risk of COVID-19 exposure, unpredictable work shifts and shifting health care policies, and worry over family member risk. This frontline health care population provides a unique example to test the feasibility of detecting stress using wearable-based technology and smartphone apps. Further, this population could inform understanding of how stress impacts susceptibility to infection, given the damaging impact of stress on our immune system [19]. The accurate measurement of stress responses in real time and in naturalistic settings has so far been a challenge [20], limiting our understanding of how different facets of acute or sustained stress increases susceptibility to breakdown and disease. Studies of stress in frontline populations exist [21], but focus on self-reported stress during aperiodic frequencies, as opposed to high-resolution approaches using digital technologies. Further, studies aimed at objective stress detection using sensor-based tools, irrespective of population, are typically limited to controlled experimental settings (eg, [22]). There are few studies applying wearable technologies used in real-world settings for the detection of stress. Among the few pilot studies that have been conducted [20,23-25], wearable technologies are showing promise for detecting shifts in health status, stress, and well-being across different populations.

The COVID-19 pandemic reflects a unique natural experimental condition where frontline workers were exposed to substantial stress beyond that already present in their pre–COVID-19 day-to-day work environment. Their on-shift time provided a naturally occurring “stress on” period, while their off-shift time provided a “stress off” period and an opportunity to follow an individual’s recovery from stress. The aims of the Stress and Recovery Study were to:

- Assess the feasibility of a participant-centric digital approach to collect both participant-reported subjective and objective measured longitudinal high-resolution data on immediate stress responses, intermediate signs of stress, recovery from stress, and COVID-19 infectivity by engaging frontline health care workers in the use of digital sensors
- Determine the feasibility of detecting and tracking changes in immediate, intermediate stress, and recovery from stress in frontline health care workers working with patients in the COVID-19 pandemic environment

## Methods

### Overview

**Figure 1:**
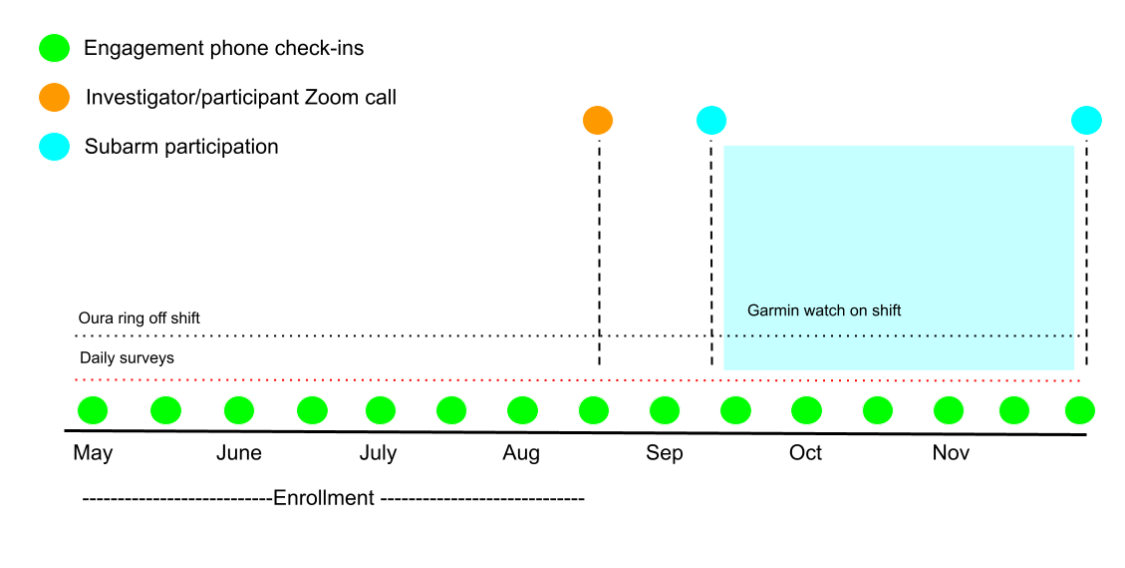
Stress and Recovery study design.

This study involved engaging US frontline health care workers from a variety of locations who were either working directly with patients with COVID-19 or whose work routines were shifted as a result of the COVID-19 pandemic. This study used a participant-centric design [16-18] (Figure 1) that comprised three primary components and a follow-up period of 4 to 6 months: (1) patient-reported active and passive assessments of stress and symptoms from a smartphone app; (2) objective measured assessments of acute stress from wearable sensors; and (3) a participant codriven engagement strategy that centered on providing knowledge and support involving regular check-in phone calls with study staff, using participant feedback in real time to improve and fine-tune the research protocol to improve participant experience, and the addition of new study subarms. A virtual event was held where researchers and participants who wished to join were able to engage in an online group setting (anonymously) halfway through enrollment. This study was approved by the Institutional Review Board, Advarra (4UCOVID1901, Pro00043205) and was registered with ClinicalTrials.Gov (NCT04713111).

### Recruitment and Onboarding

Frontline health care workers were recruited from May to August 2020. A multipronged recruitment approach was developed that involved engaging trusted leaders from organizations and sites with high outreach to our target population (eg, American Association of Critical Care Nurses), engaging supervisors at selected health care institutions, and a social media campaign (eg, Facebook, Twitter, and Linkedin). Tailored workplace-specific recruitment materials were developed and, during the enrollment period, assessed in real time how participants were learning about the existence of the study to understand the most effective recruitment strategies. Recruitment materials were distributed through workplace-specific newsletters and websites, and through our social media channels.

### Population

Frontline health care workers were invited to participate including medical doctors, doctors of osteopathy, physician assistants, registered nurses, advanced practice registered nurses, and other allied health care workers. Inclusion criteria were must be working directly with patients with COVID-19, slated to do so within the next 2 weeks, or work routines have been moderately or extremely impacted by the COVID-19 pandemic; older than 18 years; able to speak, write, and read English; able to provide informed consent; no known SARS-CoV-2 current or past infection; and must own a personal iOS mobile phone (OS11 and above) with willingness to download and use the study apps and sync phone with all study sensors.

### App-Based Data Assessments

Active study data were collected and managed in a REDCap (Research Electronic Data Capture [26,27]) database, hosted and managed by the Center for International Emergency Medical Services [28]. REDCap is a secure, web-based software platform designed to support data capture for research studies. The MyCap app interface leverages REDCap and was used to produce a study app for the collection of participant-reported active data. Participants were instructed to download the MyCap app from the app store and register with the MyCap app through a unique QR Code provided to them by study staff.

Via the study app, participants were prompted to complete daily, weekly, every 2-week, monthly, and one time measures that involved sociodemographic factors, self-reported measures of daily perceived stress, intermediate signs of stress (sleep, mood, and cognition), additional health-related symptoms, influenza-like illness, individual characteristics, and cognitive active tasks (see Multimedia Appendix 1, Table S1 [29-51]). For the first half of the study ResearchKit’s active tasks [29] (Trail Making, Reaction Time, and Spatial Span Memory) were used every day, rotating the tasks each day. For the second half of the study these were replaced with Cambridge Cognition’s active tasks [30,31] (emotional bias test, psychomotor vigilance, and n-back test) rotating every other day. The study intended to include the Cambridge Cognition active tasks from the outset; however, their implementation was delayed while establishing the technical integration with the MyCap app.

The study sample was initially limited to iOS users because of anticipated nonfunctionality with the ResearchKit apps but intended to include Android users when shifting to the Cambridge Cognition tasks. However, upon pilot-testing the REDCap app with the first set of enrolled Android users, it was found that the app itself had compatibility issues. Therefore, a subset of 12 Android users enrolled in the study who did not participate in the app-based assessments.

The daily burden for completing app-based assessments was estimated at an average of 5 minutes per day (minimum daily burden 3.5 minutes, maximum daily burden 8.5 minutes), with some daily tasks taking longer and other days shorter, which depended on the cadence of the one time, weekly, every 2 week, or monthly measures. This estimate was calculated from the expected task length, not from timed participant data. A schedule was produced that spread out the one-time measures on different days within the first month, while weekly and every 2-week tasks were scheduled on different days to balance the daily burden.

Participants were given the option to download two third-party apps as part of their participation: HealthMode Cough app and RescueTime. The cough app was used to capture momentary cough during study follow-up and RescueTime as a measure of screen time (eg, time spent in and category of apps) as a proxy for objective stress and mood.

### Wearable Assessments

Participants were mailed an Oura smart ring. The Oura Ring 2 [52] is made of a light durable titanium shell and includes a temperature sensor, a gyroscope, a 3D accelerometer, and an infrared optical pulse sensor. There was a one-time setup process where the participant synced their ring to the Oura smartphone app that they were instructed to download. Throughout the study, the participant was instructed to open the Oura smartphone app to sync data off the ring over Bluetooth. The sensor collects a variety of nighttime data streams such as heart rate, heart rate variability, and objective sleep quality measures (Multimedia Appendix 1, Table S1). Participants were provided with their own symptom summaries via the Oura app. Participants were instructed to wear the ring only while off shift owing to potential infectivity risks generally associated with the ring wearing while at work in health care settings, which was especially relevant as participants were actively working with patients. This did not meaningfully hamper relevant data collection because we were most interested in measuring parameters associated with recovery from stress while participants were off shift. It was expected that the daily burden associated with using sensors, remembering to charge them, and working through sensor issues to be approximately 2 to 4 minutes per day.

### Engagement

The study engagement strategy centered on providing information and support to participants while engaging them as codrivers of the research. This strategy had two aims: (1) to engage participants in the use of the study digital devices for optimal adherence and (2) to engage participants in the design of the study. Information was provided to participants through enabling insights into measurements of health through the study sensor apps and discussing this with participants in terms of how to interpret this information and what could be learned from it. Support was provided in a variety of channels through the biweekly check-ins, listening to participant feedback and making real-time protocol changes, and providing a variety of tools or resources in an attempt to give back (online resources for stress management) and stress-reducing tools. For example, participants were offered a YELL-IT tool where they could call a number and leave an anonymous voice message of their choice that could include any release that might offer benefit (ranting, yelling, journaling, etc). The records of these phone calls were immediately programmatically deleted, and the voice messages themselves were not recorded.

### Check-in Calls

Research staff labelled as *engagement specialists* contacted study participants every week for the first month of study participation and every 2 weeks thereafter until study completion. Engagement specialists had clinical research backgrounds and experience with working with participants. These study staff were trained in the use of digital technologies and served the role to support and engage participants in their digital experience. The check-in calls with engagement specialists served three purposes: (1) to support participants in their study participation, troubleshoot technical problems, and build rapport; (2) to discuss, understand, and collect information on study experience; and (3) to discuss, understand, and collect information on study exposure and outcome information, which in this context was the experience of stress from working on the frontline in the COVID-19 pandemic environment. The check-in calls served as a venue to gather deep insights about the participant experience in general, specifically around interacting with digital sensors for stress and health tracking. Engagement specialists reviewed adherence data prior to check-ins to probe participant-specific study challenges. Check-in calls were expected to range in time depending on the need of the participant, but the initially allotted time was up to 60 minutes for each of these calls (see Results section).

Engagement specialists conducted exit interviews by phone at the end of the study; interviews lasted approximately 1 hour. Participants were asked open-ended questions relating to their work in the pandemic environment and about features of the study that might help them and others in the future.

### Addition of New Optional Subarms

Halfway through enrollment participants were invited to participate in 1 to 3 new study subarms. The subarms were announced during the joint investigator and participant Zoom (Zoom Video Communications, Inc) meeting and during biweekly check-ins. These included an arm with a wearable smartwatch to be worn on shift, an arm with a lifestyle intervention, and an arm where hair cortisol was measured. Interested and eligible participants were sent a new REDCap link to consent to participate in these subarms.

For the wearable arm, participants were provided with Garmin Vivoactive 4 smartwatches [53] and were instructed to wear these continuously for 4 weeks (including while on shift) to capture on-shift objective measures of stress.

For the intervention arm, participants were able to self-select into a physical activity subarm or a meditation subarm for 4 weeks. Participants in the meditation arm were provided a free subscription to the Headspace app—a publicly available mindfulness app that offers guided meditation sessions among many other features aimed at improving mood, sleep, and stress. Participants were instructed to complete three to five or more mindfulness sessions per week. Participants in the physical activity subarm were provided with a resource comprising a variety of free online fitness classes and were instructed to engage in 30 minutes to 1 hour of physical exercise of their choice, 2 to 3 times a week or more.

For the hair cortisol arm, participants were sent hair sample collection kits with instructions to self-collect and send a hair sample back to the study team to provide a biological measure of chronic stress during the study period [54]. Cortisol concentrations were extracted using a standard kit (ie, ELISA) by the laboratory services at the School of Nursing at the University of Washington (please see Multimedia Appendix 1, Methods for a detailed description of the subarms).

### Joint Participant-Investigator Video Meeting

A joint participant-investigator Zoom call meeting halfway through enrollment was held. The purpose of this meeting was to give participants a chance to meet the study team in person (virtually), ask questions, and give feedback. The study team gave study updates on progress and introduced the new optional study arms. Participants’ confidentiality was maintained by using anonymous mode features of the video call platform. Although participants were anonymous in the call, they could all see and hear the study team, and could participate via the chat feature to ask questions and give feedback.

### Learning by Doing

The goal of the engagement approach was to change the participant experience from feeling like only a source of data, or a blinded “subject,” to a supported project codriver and partner in the research. As this was a feasibility study, we engaged participant feedback from the study’s start and implemented protocol changes during follow-up. Accordingly, participants directly helped shape the nature of how we asked app-based assessments, how we explained study-related details, and how we will design future remote digital health studies. Participants were invited to be coauthors of study-related published work.

### Total Participation Effort

It was estimated that the total effort for study participation was on average 10 to 15 minutes per day. Beyond app-based assessments, that on average took 5 minutes per day, additional activities included charging sensors, daily opening of the study apps and syncing of sensors, viewing the data from the associated apps, miscellaneous tech issues, check-in calls, and correspondence with an engagement specialist to schedule check-in calls and other study-related activities including exit interviews and the Zoom call. This time estimate was derived by study investigators and staff communication with participants on all daily activities as previously described.

### Compensation and Benefit

Participants were not offered any monetary incentives, nor were rewards in the form of points provided for study participation. However, participants were allowed to keep the Oura Ring and the Garmin smartwatch (in subarm participants) at the end of their participation.

### Analysis

Univariate descriptive analyses of cohort characteristics, retention, and adherence are reported. Survival probabilities using the Kaplan-Meier approach were calculated to display retention over the course of the study. Bivariate associations between cohort characteristics and adherence rates were conducted using chi-square, Fisher exact (for cell counts less than five), and *t* tests where appropriate. Mixed effects linear models were used to estimate changes in weekly mean adherence rates by group status using an autoregressive covariance matrix. Thematic study insights from qualitative data are described from participant and study staff feedback. Analyses were conducted using SPSS version 27 (IBM Corp) [55].

Retention was defined as completing the minimum follow-up, which was 4 months, or retained until the end of study, which was defined by a specific cutoff date. Adherence was defined as the number of tasks completed over the total number of available tasks that could be completed by participants’ unique study time. For example, a participant with a total study time of 140 days (20 weeks) and having completed 100 daily surveys, 17 weekly surveys, and any Oura data upload (even if a partial day) for 130 days would have an adherence rate of 71.43% (100/140) for daily assessments, 85.00% (17/20) for weekly assessments, and 92.86% (130/140) for Oura wearable data, respectively.

## Results

### Description of the Cohort

The final study sample included 365 participants who enrolled in and started the study (Multimedia Appendix 1, Figure S1). The median age was 33 (range 20-67) years (Table 1). The majority of participants were female (n=325, 89.04%), White (n=302, 82.74%), and were registered nurses (n=325, 89.04%; Table 1). Participants were followed for a median follow-up of 112 (range 1-170) days during the period from May 1, 2020, to November 20, 2020. Primary reasons for exclusion were being an Android user, prior COVID-19 infection, and no direct patient care (Multimedia Appendix 1, Figure S1). Participants were located across 27 different US states, with the majority of participants working and residing (at the time of participation) in Washington (n=103, 34.68%), Minnesota (n=66, 22.22%), Massachusetts (n=38, 12.79%), Arizona (n=27, 9.09%), and Wisconsin (n=19, 6.34%). There were five reported cases of COVID-19 during study follow-up among the study participants.

**Table 1:** Characteristics of the cohort among those who enrolled, completed the study, and did not finish.

			Enrolled and started the study (N=365), n (%)		Retained^a^ (n=297), n (%)		DNF^b^ (n=68)^c^, n (%)		*P* value (retained vs DNF)	
**Age (years)**										.16^d^
	18-25	47 (12.88)		37 (12.46)		10 (14.71)				
	26-35	168 (46.03)		129 (43.43)		39 (57.35)				
	36-45	78 (21.37)		67 (22.56)		11 (16.18)				
	>46	72 (19.73)		64 (21.55)		8 (11.76)				
**Gender**										.85^d^
	Female	325 (89.04)		264 (88.89)		61 (89.71)				
	Male	40 (10.96)		33 (11.11)		7 (10.29)				
**Race**										.35^e^
	White	302 (82.74)		242 (81.48)		60 (88.24)				
	Black or African American	8 (2.19)		8 (2.69)		0 (0.00)				
	Asian/Pacific Islander	27 (7.40)		22 (7.41)		5 (7.35)				
	Native American or American Indian	2 (0.55)		1 (0.34)		1 (1.47)				
	More than one race	21 (5.75)		19 (6.40)		2 (2.94)				
	Unknown/not reported	5 (1.37)		5 (1.68)		0 (0.00)				
**Ethnicity**										.69^e^
	Hispanic or Latino	12 (3.29)		9 (3.03)		3 (4.41)				
	Not Hispanic or Latino	343 (93.97)		279 (93.94)		64 (94.12)				
	Unknown/not reported	10 (2.74)		9 (3.03)		1 (1.47)				
**Occupation**										.39^e^
	Registered nurse	325 (89.04)		264 (88.88)		61 (89.71)				
	Medical doctor	5 (1.37)		5 (1.68)		0 (0.00)				
	Medical assistant	10 (2.74)		7 (2.36)		3 (4.41)				
	Emergency medical services	2 (0.55)		1 (0.34)		1 (1.47)				
	Other^f^	23 (10.96)		20 (6.73)		3 (4.41)				

^a^Retained includes completing follow-up of 4 months or by study end cutoff date.

^b^DNF: did not finish.

^c^DNF includes participants lost to follow-up, dropped out, or withdrawn.

^d^Pearson chi-square tests.

^e^Fishers exact tests.

^f^Other occupations include social workers, respiratory therapist, surg/cardio tech, dentist, registered dietitian, or medical student.

### Recruitment

Upon screening, participants were asked “How did you hear about our study?” Of participants screened and enrolled into the study, over 50.00% (n=200) found out about the study through a recruitment email from their workplace department or floor, while approximately 40.00% (n=151) found out about the study through word of mouth. The remainder (<10.00%) found out about the study through national associations and workplace-specific newsletters and social media channels.

### Retention

**Figure 2:**
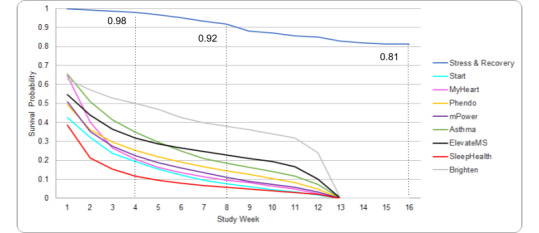
Survival probability of retaining in the study. Additional data from Pratap et al [6]. Kaplan-Meir survival curves for the Stress and Recovery study and for 8 additional digital health app-based studies as described in Pratap et al [6]. Please interpret with caution. The survival probabilities from the 8 studies included in Pratap et al [6] included a mix of different study populations some including chronic disease populations and some healthy populations with different study durations.

Of the 365 enrolled participants, 81.37% (n=297) completed the study. Of the 68 participants who did not complete the study, 11.76% (n=8) were withdrawn (study team withdrew) due to no longer meeting inclusion criteria such as no longer working with patients, furloughed, or lost study sensors; 41.18% (n=28) dropped out (participant decided to end participation) due to reasons such as no longer interested, not enjoying the study or study sensor, or too much time commitment; and 51.47% (n=35) were lost to follow-up (could not be reached on recontact). The probability of retaining in the study for 1 month was 98.00%, while the probability of retaining in the study halfway through study time was 92.00% (Figure 2). Retention for the three study subarms can be found in Multimedia Appendix 1, Figure S2.

Sample characteristics were similar in participants who started the study compared to those retained in the study. There were higher proportions of younger individuals not completing the study compared to those retained, although this was not statistically significant (*P*=.16). Other sample characteristics were similar in participants who did not complete the study compared to those retained although cell counts were low across categories (Table 1).

### Adherence

Adherence calculations are presented for those participants who completed the study (Table 2). Although initially excluded, 12 Android users were enrolled when the study protocol switched to Cambridge Cognition tasks from ResearchKit’s active tasks because of expected sensor compatibility. However, owing to troubleshooting problems with the MyCap app, these 12 individuals (minus 1 participant who did not complete the study) were unable to use the study app and were excluded from app-based adherence calculations.

**Figure 3:**
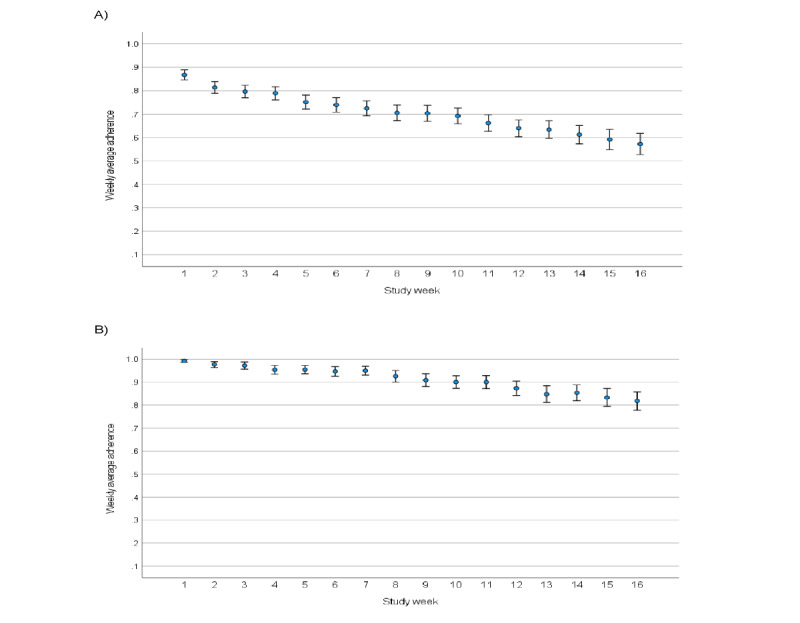
Weekly average adherence and standard errors for daily app-based tasks (A) and Oura Ring use (B) in retained participants (n=297).

Participants adhered to wearing the Oura smart ring and the Garmin smartwatch for an average of 90.60% and 90.42% of study time, respectively. App-based daily, weekly, every 2 weeks, and monthly surveys were completed on average 69.18%, 68.37% (range across different tasks 64.44%-71.86%), 72.86% (range across different tasks 72.42%-73.30%), and 68.82% (range across different tasks 68.05%-69.82%) of study time, respectively (Table 2). Every 2-week check-in phone calls were completed for an average of 75.62% of study time (Table 2 and Figure 3). The average check-in call length was approximately 14.5 minutes and ranged from 2 to 70 minutes. Measures scheduled on Fridays and Saturdays had consistently lower adherence compared to other days of the week. Average adherence was higher for the ResearchKit active tasks (80.59%) compared to the Cambridge Cognition tasks (56.49%). However, the ResearchKit tasks were integrated within the study app and were shorter in duration, while the Cambridge Cognition tasks had to be completed via an external web-based link, which may have contributed to lower adherence on these tasks. Weekly average adherence on daily app measures across age categories were similar, and not statistically significant (*F*_3_=0.20; *P*=.89), although there was a trend where higher age categories demonstrated higher adherence on Oura Ring use (*F*_3_=2.49; *P*=.06). Adherence across other sample characteristics was not explored owing to small sample sizes across categories.

Average adherence in the week after the joint participant-investigator Zoom call was held showed large increases for app-based daily surveys (82.93%), for Cambridge Cognition tasks (88.97%), and in Oura Ring use (97.89%).

**Table 2:** Adherence rates by study activity.

	Full study period
Study app surveys, n	286^a^
Daily survey^b^, %	69.18
Weekly surveys, %	68.37
Biweekly surveys, %	72.86
Monthly surveys, %	68.82
ResearchKit tasks, n^c^	164
Cognitive active tasks, %	80.59
Cambridge cognition, n^d^	289
Cognition tasks, %	56.49
ES^e^ check-ins, n	297
Biweekly check-ins^f^, %	75.62
Oura Smart Ring, n^g^	296
Oura Ring use, %	90.60
On-shift wearable subarm, n	95
Garmin Smartwatch use, %	90.42

^a^Excluding 11 participants who were Android users and unable to use the study app.

^b^All study app survey completion calculations exclude 12 participants (11 retained) with Android sensors who have no study app data

^c^ResearchKit active tasks were switched to Cambridge Cognition tasks on July 6, 2020; therefore, some participants did not receive at least 1 ResearchKit tasks as reflected by a smaller study sample size

^d^The higher sample size reflects a few Android users who were able to access the web-based Cambridge Cognition links.

^e^ES: engagement specialist.

^f^Of the 297 retained participants, 2 completed 0 check-ins.

^g^One retained participant never synced their ring to the app.

### Engagement Impact on Adherence

Although this study was not designated to explicitly test different aspects of the engagement approach on study acceptability and experience, to explore whether study-related events had an impact on adherence, weekly average adherence on active daily app-based surveys in participants enrolled prior to the joint participant-investigator Zoom call, and therefore had the opportunity to participate (n=246), and participants enrolled after the Zoom call, and therefore did not participate (n=39), were calculated. As seen in Multimedia Appendix 1, Figure S3, participants who were enrolled later in the enrollment period and did not have the opportunity to participate in the joint investigator-participant Zoom call showed lower weekly average adherence (mean 0.66, SD 0.22) on daily app-based tasks compared to those enrolled prior to this meeting (mean 0.71, SD 0.21); although, this was not statistically significant over study time (*F*_1_=3.06; *P*=.81; Multimedia Appendix 1, Table S2).

### Learning by Doing: Insights Learned During Study Follow-up

Knowledge was gained from the check-in calls with engagement specialists and from the engagement specialists themselves through discussions with participants that fueled insights into study improvements. The insights learned can be found in Multimedia Appendix 1, Table S3. Protocol changes implemented can be found in Multimedia Appendix 1, Table S4. Key themes centered on were privacy (particularly on the surveillance nature of some app features (eg, RescueTime), usability, perceived utility, and knowledge of how to interpret sensor readouts, particularly the objective data (eg, heart rate and heart rate variability). Dedicating check-in calls to explain the purpose of individual measures and how data are used by third-party apps helped with these challenges. Although not an initial purpose of check-in calls, in discussing stress and symptom experience with participants, these calls also served as an outlet for some participants to discuss with someone outside their place of employment their COVID-19 frontline experience. Accordingly, these calls may have produced an inadvertent interventional effect.

## Discussion

### Main Findings

This study tested the feasibility of a participant-centered digital health study with a daily burden of 10 to 15 minutes in frontline health care workers. We found support for the feasibility and acceptability of this approach with 81% (n=297) retention, while average adherence for wearable sensor use and daily app-based assessments was approximately 90% and 70% of study time, respectively. This contrasts to typical reported retention and adherence rates in digital medicine studies that tend to be lower than 50% [6] and that had much lower daily burdens, although the underlying populations of these studies are different, which makes direct comparisons difficult. In addition to high retention and adherence, the collection of large volumes of objective data alongside contextual self-reported subjective data was able to collect what is often missing from the *light touch* digital health study.

The COVID-19 pandemic has highlighted the increasing importance of being able to operate, communicate, and conduct research remotely and digitally. Yet, historically remote digital studies have been hampered by low retention and engagement of participants. This poses obvious challenges for the usability and generalizability of digital data and is also an early warning signal. Poor engagement at the research phase provides clues into the challenges we will face at the health care implementation phase. The engagement approach developed here involved three components: (1) supportive check-in calls with engagement specialists during follow-up, (2) a learning-by-doing approach that leveraged direct feedback from participants collected during check-ins to fuel real-time study improvements and participant experience, and (3) new study features and a virtual investigator-participant event. Findings from this feasibility study suggest these patient-centered strategies offer enough value to sufficiently engage participants without monetary/reward-based incentives. Although participants were offered to keep their Oura Ring and Garmin watch at the end of participation, no monetary incentives or point-based rewards systems were used. Both the increase in adherence after the joint participant-investigator Zoom call and higher daily adherence among those with the opportunity to participate in the Zoom call suggests that potential self-awareness and benefit from the sensors alone are not the only reason for sustained adherence to protocols. Further, this approach enabled the collection of rich objective measured data alongside participant self-reported subjective data. The common *light touch* digital health study often lacks adequate contextual self-reported subjective data to ground and validate the objectively collected information, which poses challenges in interpreting the data. These findings suggest that engaging participants in the appropriate way can enable a high burden study and the subsequent collection of needed contextual self-reported subjective data.

Digital sensors that can return symptoms back to the user enable a two-way learning experience in which participants learn about themselves in real time and provide insights to researchers, in contrast to traditional methodologies that collect data from participants in blind or shielded ways, where the data are then unveiled at the end of the study. The former enables accelerated learning at the pilot research phase: a learning-by-doing approach that leverages digital tools to enable participants to actively partake in and shape their digital research experience through tracking their own health data and discussing these data with study investigators in real time. However, the impact of tracking objective measures of health is largely unknown in terms of how being enabled to track personal objective data impacts the user. Further, wearable sensor companion apps have embedded nudges and prompts to shift behavior based on the collected data. Some participants noted frustration with both the returning of the Oura Ring collected symptom summaries and associated feedback prompts, as these were nonactionable, particularly in a health care professional population. On the contrary, others viewed these returned symptoms and nudges positively. Commonly, participants noted a desire to have more knowledge about interpreting the sensor readouts and were curious about other participants’ data. Beyond digital literacy, there is complex knowledge and support required in the use of digital tools for stress and health tracking. The field is in an early phase of understanding how individuals from diverse populations will interact with digital technologies at home and in everyday life that will be needed for the successful implementation of digital approaches into health systems and for their use in transforming individualized care. Support in the use of these tools is a current gap. The notion of a *digital expert* or counselor, not dissimilar to genetic counselors and like the engagement specialists used here, may be one approach to bridge this gap both for digital research and digital health care.

### Limitations

The study population included predominantly White health care professionals who are nonrepresentative of non-White and non–health care provider populations in having higher than average knowledge of research and higher health literacy. It is unclear how the engagement approach might generalize in other populations. On the other hand, this population is a busy, high-stress population. In light of this, one could argue that this particular population would be difficult to engage in a high-friction study owing to work-life constraints. A nonprobability sample was included; therefore, selection biases may be present where participants enrolled may be more likely to engage or have interest in the use of wearable sensors for health tracking compared to those who were uninterested. A control group of participants who did not receive the adjacent engagement strategy were not included; therefore, we cannot imply causality of this approach or parse the different possible drivers of success on retention and adherence results. Further, the cost of the Oura Ring and Garmin Smartwatch is high (>US $500). Although no monetary incentives were offered, the opportunity to keep these devices could have impacted willingness to retain and adhere to protocols.

The participant-centered learning-by-doing approach used here worked well for a feasibility study where the primary purpose is to learn about how well a methodology or a tool will work for health-related purposes. However, these approaches may not be well suited to other types of studies that require more controlled data collection such as in randomized controlled trials. Enabling participants to track their own health outcomes while they are under study may increase risk of bias in the controlled study context through participants’ awareness of symptoms, particularly through companion app nudges and prompts. Although, this hallmark challenge in traditional controlled studies largely reflects a risk to altering perceptions of the outcome of interest, which is how symptoms are traditionally measured. In the context of enabling individuals to be aware of their own measured objective signs of stress and disease, the importance of this traditional challenge becomes less clear. An additional possibility is that the implemented engagement approach may have produced a positive interventional effect from either the returned symptoms or the every 2-week check-in calls, which could modulate the stress signal in the data.

Additional challenges of this approach encompass a time burden on researchers and staff. Both investigators and staff were highly engaged during the study follow-up. Each check-in call took on average 15 minutes, with some calls taking as long as an hour. However, in the context of traditional research, where research staff conduct in-person assessments and manually enter data, it remains unclear how much extra time burden this approach actually produces. This learning-by-doing approach may also be difficult to implement depending on research ethics board review timelines. The institutional review board used here enabled rapid review of modifications enabling quick and efficient amendments to the protocol. Finally, this approach may not be scalable for large digital health studies, and it is unclear how effective it might be in other populations.

### Future Directions

Future work should test different aspects of these types of digital engagement approaches in controlled settings, including control groups of financial incentives only and no incentives, to further determine their effectiveness across different populations. Further, the impact (benefits and harms) of returning objective measures of health requires more interrogation. As for increasing understanding of stress detection from wearable-based technologies, other researchers are encouraged to access the Stress and Recovery data that will be hosted on the Synapse at Sage Bionetworks (available in December 2021) to progress this field.

### Conclusions

Digital technologies could facilitate a new era of participant-driven models in research and medicine [56]. As datafication [57], the process of digitizing most aspects of human life, continues to intensify, the need to incorporate insights from the individuals who are the source of those data grows increasingly important. A common shortcoming of the *light touch* digital health study is lacking adequate ground-truth data in the form of participant-reported subjective information. Given the early state of this field, ground truths to validate measurements of health are important yet are so often missing. Although statistical power is important, it is unclear whether an increase in the study size can counter the benefits seen from the depth of collecting additional contextual information. Incorporating a learning-by-doing digital approach facilitates a closed-loop research process whereby participants can offer rich self-reported context for objectively measured data, while researchers can learn in real time and offer knowledge and support back to participants. For this to work, trust and respect between participant and researcher is essential, as it should always be and could serve as a model to be leveraged for the implementation phase of digital medicine.

## Appendix

Multimedia Appendix 1 Supplemental information.

## Abbreviations

REDCap: Research Electronic Data Capture
