# Correction: An Alternative to the Light Touch Digital Health Remote Study: The Stress and Recovery in Frontline COVID-19 Health Care Workers Study

**DOI:** 10.2196/38188

**Published:** 2022-04-18

**Authors:** Sarah M Goodday, Emma Karlin, Alexandria Alfarano, Alexa Brooks, Carol Chapman, Rachelle Desille, Shazia Rangwala, Daniel R Karlin, Hoora Emami, Nancy Fugate Woods, Adrien Boch, Luca Foschini, Mackenzie Wildman, Francesca Cormack, Nick Taptiklis, Abhishek Pratap, Marzyeh Ghassemi, Anna Goldenberg, Sujay Nagaraj, Elaine Walsh, Stress and Recovery Participants, Stephen Friend

**Affiliations:** 1 4YouandMe Seattle, WA United States; 2 Department of Psychiatry University of Oxford Oxford United Kingdom; 3 Nationwide Children's Hospital Columbus, OH United States; 4 MindMed New York, NY United States; 5 Tufts University School of Medicine Boston, MA United States; 6 Dalla Lana School of Public Health University of Toronto Toronto, ON Canada; 7 School of Nursing University of Washington Seattle, WA United States; 8 Evidation Health Inc San Mateo, CA United States; 9 Cambridge Cognition Cambridge United Kingdom; 10 Department of Psychiatry University of Cambridge Cambridge United Kingdom; 11 Krembil Center for Neuroinformatics Center for Addiction and Mental Health Toronto, ON Canada; 12 Vector Institute Toronto, ON Canada; 13 University of Washington Seattle, WA United States; 14 King's College London London United Kingdom; 15 Institute for Medical Engineering and Science MIT Cambridge, MA United States; 16 Department of Electrical Engineering and Computer Science MIT Cambridge, MA United States; 17 The Hospital for Sick Children Toronto, ON Canada; 18 Department of Computer Science University of Toronto Toronto, ON Canada; 19 Canadian Institute for Advanced Research Toronto, ON Canada

In “An Alternative to the Light Touch Digital Health Remote Study: The Stress and Recovery in Frontline COVID-19 Health Care Workers Study” (JMIR Form Res 2021;5(12):e32165), the authors noted one error.

In the originally published article, author Shazia Rangwala's name was inadvertently not included in the list of authors. They have been added as the 7th author and with the first affiliation. The list of authors and affiliations now appears as follows:

*Sarah M Goodday^1,2^, PhD; Emma Karlin^1^, MPH; Alexandria Alfarano^3^, MA; Alexa Brooks^1^, MSc, RD; Carol Chapman^1^, MPH; Rachelle Desille^1^, BA; Shazia Rangwala^1^, MPH; Daniel R Karlin1,^4,5^, MA, MD; Hoora Emami^6^, BA; Nancy Fugate Woods^7^, BSN, PhD, FAAN; Adrien Boch^8^, MSc; Luca Foschini^8^, PhD; Mackenzie Wildman^8^, PhD; Francesca Cormack^9,10^, PhD; Nick Taptiklis^9^, BA; Abhishek Pratap^11,12,13,14^, PhD; Marzyeh Ghassemi^12,15,16^, PhD; Anna Goldenberg^12,17,18,19^, PhD; Sujay Nagaraj^12,17^, BSc; Elaine Walsh^7^, PhD, RN, PMHCNS-BC; Stress and Recovery Participants^1^; Stephen Friend^1,2^, MD, PhD*

*^1^4YouandMe, Seattle, WA, United States*

*^2^Department of Psychiatry, University of Oxford, Oxford, United Kingdom*

*^3^Nationwide Children's Hospital, Columbus, OH, United States*

*^4^MindMed, New York, NY, United States*

*^5^Tufts University School of Medicine, Boston, MA, United States*

*^6^Dalla Lana School of Public Health, University of Toronto, Toronto, ON, Canada*

*^7^School of Nursing, University of Washington, Seattle, WA, United States*

*^8^Evidation Health Inc, San Mateo, CA, United States*

*^9^Cambridge Cognition, Cambridge, United Kingdom*

*^10^Department of Psychiatry, University of Cambridge, Cambridge, United Kingdom*

*^11^Krembil Center for Neuroinformatics, Center for Addiction and Mental Health, Toronto, ON, Canada*

*^12^Vector Institute, Toronto, ON, Canada*

*^13^University of Washington, Seattle, WA, United States*

*^14^King's College London, London, United Kingdom*

*^15^Institute for Medical Engineering and Science, MIT, Cambridge, MA, United States*

*^16^Department of Electrical Engineering and Computer Science, MIT, Cambridge, MA, United States*

*^17^The Hospital for Sick Children, Toronto, ON, Canada*

*^18^Department of Computer Science, University of Toronto, Toronto, ON, Canada*

*^19^Canadian Institute for Advanced Research, Toronto, ON, Canada*

The correction will appear in the online version of the paper on the JMIR Publications website on April 18, 2022, together with the publication of this correction notice. Because this was made after submission to PubMed, PubMed Central, and other full-text repositories, the corrected article has also been resubmitted to those repositories.

