# Supplementary material for: An Alternative to the Light Touch Digital Health Remote Study: The Stress and Recovery in Frontline COVID-19 Health Care Workers Study
Source: JMIR Form Res. 2021 Dec 10;5(12):e32165. doi: 10.2196/32165 (PMC8668021; doi:10.2196/32165)
Supplement: Multimedia Appendix 1 [file formative_v5i12e32165_app1.pdf]

Supplemental Material: An alternative to the ‘light touch’ digital health remote study: The Stress and Recovery in Frontline COVID-19 Healthcare Workers Study

Supplemental Table 1. Study app-based tasks

**Assessments**	**Cadence**	**Description**
Demographics and Lifestyle*	Baseline (+- 25 days)	Basic demographics, social determinants of health and lifestyle factors
Medical History and medications*	Baseline (+- 25 days)	Medical History and medications
Adverse Childhood Events (ACE)[32-34]	Baseline (+- 25 days)	The ACE questionnaire is a 10-item measure of an individual’s eperiences with early adversity occuring prior to age 18 years including physical and sexual abuse, neglect and household dysfunction. The ACE questionnaire has established validity[1] and good internal consistency (e.g.,Cronbach α=0.88)[33]
Life Events Checklist (10-item)[35]	Baseline (+- 25 days)	The Life Events Checklist is a 10-item self-reported survey that assesses an individual’s past-year significant life events and the perceived experience of these events from very good to very bad.
Post-Traumatic Symptom Disorder Civilian Checklist (PCL-C)[36,37]	Baseline (+- 25 days)	The PCL-C is a self-report rating scale for assessing the 17 DSM-IV symptoms of PTSD. The PCL-C has established validity and good internal consistency (Cronbach α=.97).[35]
Ten-item Personality Inventory (TIPI)[38]	Baseline (+- 25 days)	The TIPI is a single item with 10 response options that assess the Big 5 personality traits. This brief measure has established internal consistency: Cronbach α’s range between 0.40-0.73 across subscales.[38]
PPE and Exposure Scale*	Daily	The PPE scale is an assessment of protective equipment usage for virus exposure prevention.
Self-Assessment Mannequin (SAM)*	Daily	The SAM is a 5-point icon-based assessment of daily mood, energy, cognition, stress, cough.
Daily Stress*	Daily	The Daily Stress Measurement is a brief questionnaire assessing an individual’s daily stress experience.
CANTAB Emotional Bias Test (EBT)[30]	Every other day	Active task that detects perceptual bias in facial emotion perception
CANTAB Psychomotor Vigilance Test (PVT)	Every other day	Active task that objectively assessed fatigue-related changes in alertness associated with sleep loss, extended wakefulness, circadian misalignment, and time on task.
Cogkit N-Back Test[31]	Every other day	Active task that assesses working memory recall in an individual. It has not been validated, however is deemed useful for experimental research on working memory[31]
Healthkit Trail Making Test[29, 39]	Every other day	Active task in which an individual is asked to connect a series of numbered dots, assessing visual attention and task switching. The TMT is a validated measure with correlations between alternative forms of Trails A and BD ranging between r=.76 to r=.94.[29]
Healthkit Reaction Time Test[29, 40]	Every other day	Active task in which an individual is asked to shake their device when a blue dot appears, assessing reaction. Similar mobile-device reaction tests have been found to have high reliability and observed power of 0.96 (n = 27, α < 0.05, effect size = 0.59)[40]

Healthkit Spatial Span Memory Test[40]	Every other day	Active task in which an individual is asked to tap shapes in the order they were presented moments earlier, assessing short-term memory, known to be a useful tool in research settings[12]
Perceived Stress Scale 4 (PSS-4)[41, 42]	Weekly	The PSS-4 is a 4-item measure of an individual’s perceived past week stress. It has been validated (Cronbach α =0.79).[42]
Cough EMA/VAS*	Daily/Weekly	The EMA/VAS is an assessment of coughing frequency and severity in the past hour.
COVID-19 Baseline and Ongoing Risks*	Weekly	The COVID-19 baseline and ongoing measurement is a questionnaire on an individual’s clinic schedules & exposure levels.
PROMIS Sleep Disturbance (SD) (V1.0 6a)[43, 44]	Weekly	The PROMIS-SD is a 6-item self-reported measure of an individual’s perceptions of sleep quality, depth and restoration. PROMIS-SD has been validated with good internal consistency (Cronbach α=0.84).[43]
PROMIS Sleep Related Impairment (SRI) (V1.0 8a)[43, 44]	Weekly	The PROMIS-SRI is an 8-item self-reported measure of an individual’s perceptions of alertness, sleepiness and functional impairments. PROMIS-SRI has been validated with good internal consistency (Cronbach’s α=0.91)[43]
Fatigue Severity Scale (FSS)[45]	Weekly	The FSS is a 9-item scale that measures motor aspects of fatigue and assesses the severity this has on an individual. It is a validated and reliable measure (Cronbach’s α=0.93).[45]
Patient Health Questionnaire-9 (PHQ-9)[46]	Every two weeks	The PHQ-9 is a validated 9-item measure of an individual’s depressive symptoms over the past 2 weeks that has a sensitivity of 88% and a specificity of 88% for major depression.[46]
General Anxiety Disorder-7 (GAD-7)[47]	Every 2 weeks	The GAD-7 is a validated 7-item measure of an individual’s anxiety symptoms over the past 2 weeks (Cronbach’s α=0.92)[47]
PROMIS Emotional Support (4a)[48]	Monthly	The PROMIS-ES is a 4-item assessment of an individual’s available emotional support. The PROMIS subscales are validated with good internal consistency for all subscales (Cronbach α>0.88)[48]
PROMIS Global-10[49]	Monthly	The PROMIS Global-10 is a 10-item measure of an individual’s general health and quality of life. It has established internal consistency for global physical health (Cronbach’s α=0.81) and mental health (Cronbach’s α=0.86).[49]
Survey Response Inclination (SRI)**	Monthly	The SRI is a 1-item measure of an individual’’s survey response inclination.
RAND 36-Item Short Form Health Survey[50, 51]	Monthly	The RAND-36 is a self-reported 36-item measure of health-related quality of life that has established validity and reliability Cronbach’s α between sub-scales ranges from 0.65-0.94.[50]
Cough Audio Data***	As needed	Cough data is collected through a Smartphone App that records an individual’s cough audio when a cough has occurred

Patient-Reported Outcomes Measurement Information System (PROMIS); Ecological Momentary Assessment/Visual Analogue Scale (EMA/VAS)

*Indicates a measure developed by 4YouandMe
**Indicates a measure developed by Evidation Health, Inc.
***Indicates a measure provided by HealthMode, Inc.

Supplemental Material: An alternative to the ‘light touch’ digital health remote study: The Stress and Recovery in Frontline COVID-19 Healthcare Workers Study

Supplemental Table 2. Daily survey and Oura weekly average adherence across age categories

	Daily Surveys				Oura			
Age (years)	n	Mean	SD	*P*-value[1]	n	Mean	SD	*P*-value[1]
18-25	37	0.68	0.19	.894	37	0.85	0.20	.060
26-35	125	0.70	0.22		129	0.92	0.16	
36-45	63	0.71	0.21		67	0.89	0.15	
46+	61	0.71	0.20		63	0.93	0.13	

SD” Standard Deviation
[1]One-way ANOVA

Supplemental Table 3. Check-in insights from participants and engagement specialists

Study Feature	Insights from Participants
Study app experience	- Several themes noted by participants that would help with completion of daily tasks centered on ease of usability, highlighting the importance of integration of 3rd party applications in the central study app, non-reliance on web-based 3rd party applications and individual tailoring of the app experience (custom push notifications and survey completion windows).<br>- Keeping individual surveys short (e.g., <1 minute) was noted as important. While completing several surveys that totalled approximately 5 minutes per day was acceptable, individual surveys that were longer (3-5 minutes) were less acceptable as participants had to find longer chunks of time in their day for completion. However, a collection of short surveys that could be completed at different times was more acceptable.<br>- Privacy concerns were noted relating to third party applications of a “surveillance” nature. We found that communicating with participants in the context of why we are collecting certain data points such as total screen time from Rescue Time helped with adherence. There were some misperceptions of how these 3rd party applications use data. For example, while RescueTime requests to access location, these data are used for the app's functionality to track screen time, and remains on the users device, and is not sent to Rescue Times servers.
Study Measures	- Survey measures with non-applicable wording and repetitiveness when variation in the construct was minimal were a noted annoyance and produced participant fatigue. For example, personal protective equipment changes were initially measured on a daily basis, but from both reviewing the variation in the data, and from participant insights, this was too often and accordingly, was changed to a weekly cadence. For some measures, it is unclear whether variation at a daily level exists in unpredictable scenarios, which was the case in the early months of the pandemic. Having the capability to change these cadences may reduce annoyances for participants, and in turn improve adherence.<br>- Some participants noted consistent challenges with cognitive active tasks, while others found these tasks fun. Cognitive tasks are intended to be challenging, yet when administered at higher frequencies produce additional burden and in some participants, these tasks were felt to impact self-esteem. We found that again, communicating context to participants as to why we are collecting certain measures improved understanding.<br>- Participants expressed interest in receiving summaries of their survey data.
Wearables	- Challenges in using the study wearable devices included wearing the ring during activities, worry over losing the ring, actually losing the ring, technical issues and the want for more information on

	interpreting the returned objective data from the associated app. While there was a worry by several participants about ring loss, the actual proportion losing the ring was very small (2.7%), although this should be expected occasionally, and accounted for in costing.<br>- A technical issue at the beginning of the study with several participants was forgetting to open the associated app to sync with the wearable device. This can produce problems in data flow.<br>- Perceived utility of the objective data was questioned by some, and there was a general want for more information on how to interpret the returned objective data. There was a general interest in being enabled to track objective sleep metrics while some found these notifications from the app frustrating as they were non-actionable. For example, the Oura ring makes recommendations on pushing yourself, or taking a break based on last night's sleep quality scores and how well you have recovered. Acting on these prompts is challenging for healthcare professionals who work shift work and was a noted frustration by some. Others noted a positive experience from these notifications, suggesting this actually produced behaviour change. Taken together, the significant importance of availability of engagement experts to assist with wearable device orientation, to troubleshoot technological issues and to discuss the nature of the data captured from these devices was very evident.
Check-in calls	- In general, these check-in calls were well received and enjoyed by participants. Only 2 participants did not engage with these calls during participation. Not all participants were interested in talking on the phone and noted a preference to have the option of using text, email, and in some, video chats like Facetime.<br>- Feedback from engagement specialists indicated the exercise of reviewing participant data prior to the check-in was useful for gaining context and understanding a greater depth of participant experience.

Supplemental Material: An alternative to the 'light touch' digital health remote study: The Stress and Recovery in Frontline COVID-19 Healthcare Workers Study

Supplemental Table 4. Real-time protocol changes implemented

Study Feature	Protocol Change
Study app	- Included screening questions to daily assessment measures on work shift to reduce daily burden of shift-relevant questions.<br>- Revised the cadence of personal protective equipment measures from daily to weekly (PPE use was not dynamic enough at the daily level)
Check-in calls	- Over the course of the study, the study team worked with EEs to tailor scripts that provided participants with information that they indicated wanting to know more about. For example, what their nighttime HRV really meant, or why the RescueTime app asks to collect location data.<br>- The study team shifted the nature of questions asked on check-in calls as the study progressed in response to having less technological/troubleshooting problems with the devices and apps. The shift in nature of questions was to gain a better understanding and context around the participants' stress, and perceived sources of stress and how they were interacting with the digital devices and apps.
Wearables	- Realizing that some participants were able to wear wearable devices while on shift, and because of feedback from participants wanting to know what they're objective physiological measures looked like while at work, a new sub-arm was introduced where participants were provided with a Garmin Vivoactive 4 Smartwatch to wear while on shift.
Other	- In response to participant feedback and wanting to reduce their stress, a new lifestyle intervention sub-arm was introduced where participants were invited to participate in a four-week physical exercise, or a meditation/mindfulness arm where we provided free access to the Headspace App.<br>- A new participant benefit stress reduction tool was introduced called YELL-IT where participants could call a number and leave a voice message of their choice that could include any release that might offer them benefit.<br>- An additional sub-arm to collect a one-time hair sample to assess participants' cortisol concentrations as a proxy of they're past chronic stress was included.

Supplemental Material: An alternative to the ‘light touch’ digital health remote study: The Stress and Recovery in Frontline COVID-19 Healthcare Workers Study

Supplemental Figure 1. Study sample selection and retention

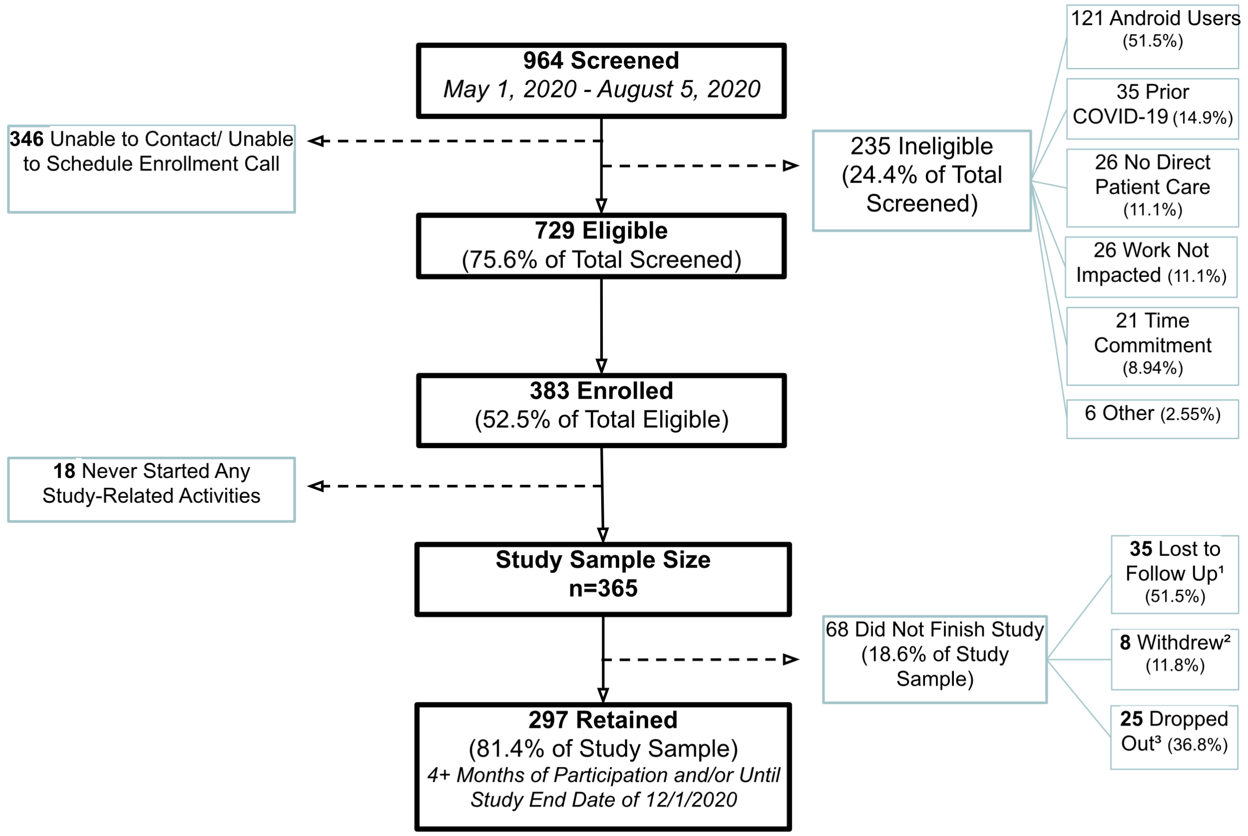

Supplemental Material: An alternative to the 'light touch' digital health remote study: The Stress and Recovery in Frontline COVID-19 Healthcare Workers Study

Supplemental Figure 2. Sub-arm study sample selection and retention

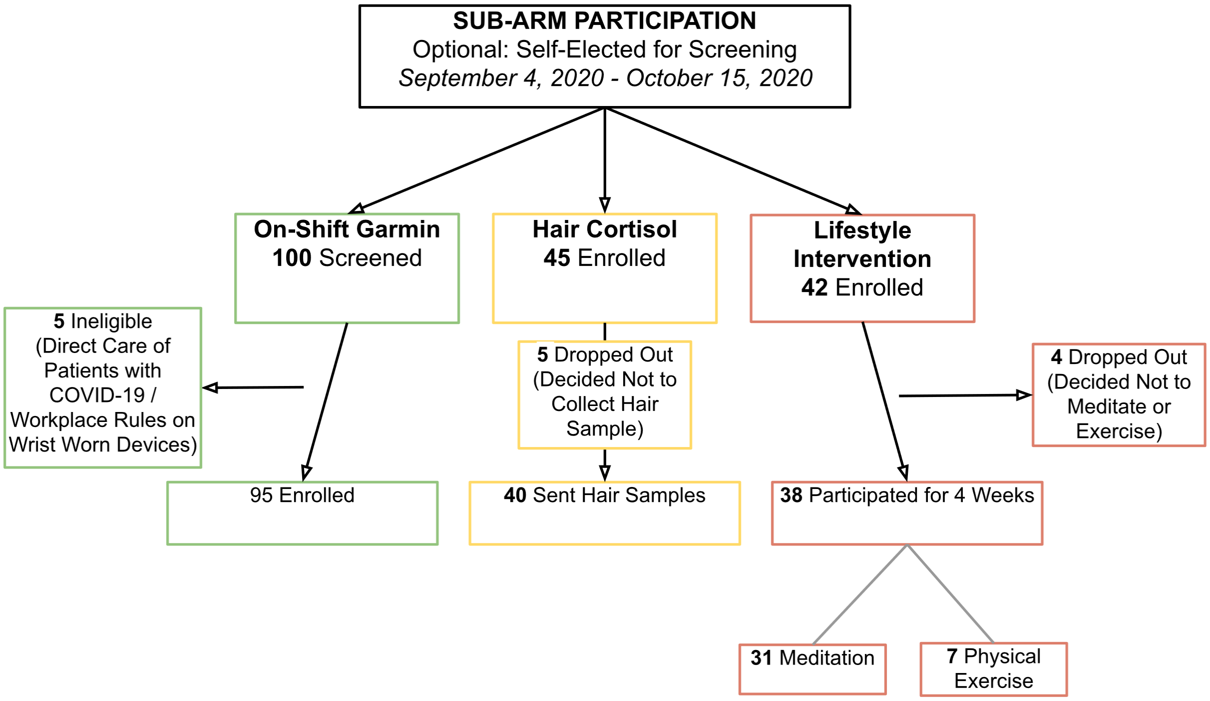

Supplemental Material: An alternative to the 'light touch' digital health remote study: The Stress and Recovery in Frontline COVID-19 Healthcare Workers Study

Supplemental. Figure 3. Weekly average adherence and 95% confidence intervals over study time for daily app-based tasks (A) and Oura ring usage (B) in those enrolled prior to the zoom call (n=246) and those enrolled after the zoom call (n=39)

A)

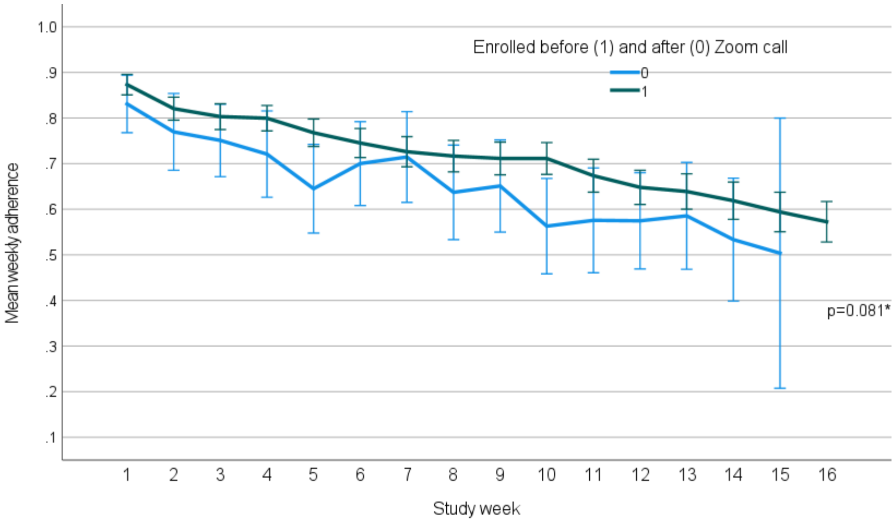

B)

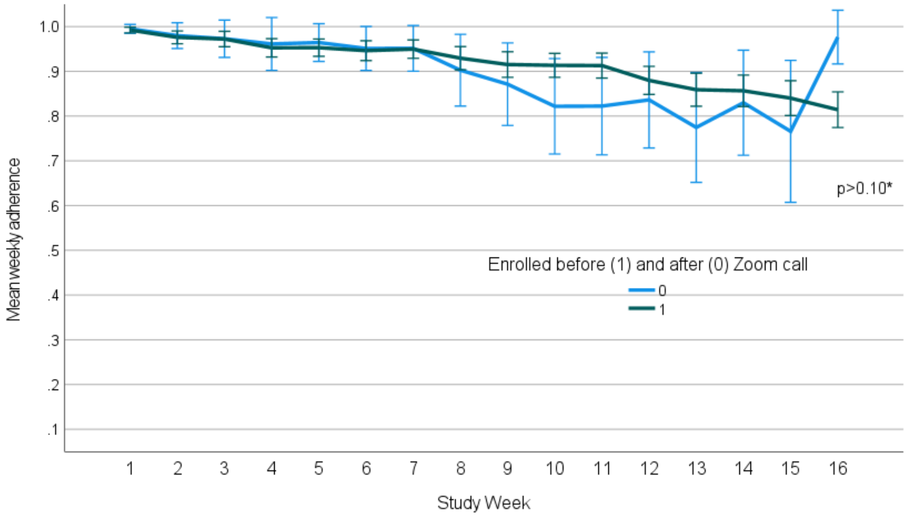

*Mixed effects linear model

Supplemental Material: An alternative to the 'light touch' digital health remote study: The Stress and Recovery in Frontline COVID-19 Healthcare Workers Study

Supplemental Methods. Additional sub-arm protocol material

Overview

Participants had the choice to participate in one or all of the following sub-arms: Lifestyle intervention, wearable on shift, and hair cortisol. The sub-arms were announced during the joint investigator/participant zoom meeting and during bi-weekly check-ins. Eligible and interested participants contacted their engagement specialist expressing interest in participating, or did so during a biweekly check-in. Interested and eligible participants were sent a new REDCap link to consent to participate in these sub-arms.

Intervention sub-arm

Purpose:

- To offer a stress alleviating intervention to participants (to give something back) and see if there is a detectable shift in stress signal in this sub-arm compared to the remainder of the sample not participating in this arm.

Inclusion criteria:

- Any currently enrolled participant expressing interest with four weeks left of study participation or is willing to extend full study participation post four months

Exclusion criteria:

- If already engaging in both inward (meditation/mindfulness-based techniques) and outward activities (physical exercise) more than twice a week.
- If engaging in only one of these activities more than twice a week, then the participant had to choose the other activity.

Procedure:

- Participants were given study instructions over the phone with their engagement specialist and were sent an instructional package via email that includes two choices of lifestyle interventions (inward and outward activities). Interested participants were offered to choose one of the interventions to partake in for four consecutive weeks.

Interventions:

- *Meditation/mindfulness and lifestyle (Inward)*
    - Participants were provided with a free subscription to Headspace https://www.headspace.com/ and were instructed to download the app from the app store. Participants were instructed to become familiar with the app and try out a few meditation sessions to find what type of sessions best suit them and were instructed to complete 3-5 or more sessions per week during the 4-week participation.

- *Physical exercise (Outward)*
    - Participants were provided with a resource comprising a variety of free online fitness classes and were instructed to engage in 30 minutes to 1 hour of physical

exercise, 2-3 times a week or more that could include an exercise of their choice (e.g., running, strength training, yoga, pilates, barre).

On shift wearables sub-arm

Purpose:

During the course of the Stress and Recovery main study we were informed by some participants that smartwatches were allowable at some workplaces. The main study was collecting off-shift objective stress data from the Oura ring, but the ring does not work during the day and is not intended for the collection of daytime objective data. We included the Garmin Vivoactive 4 smartwatch to collect on work shift objective stress data (e.g., heart rate, heart rate variability, respiratory rate and activity).

Inclusion criteria

- Participants who are ‘likely’ to not work with COVID-19 patients or have several consecutive days where they will not work with COVID-19 patients in the next 3-4 weeks
- Participant’s place of work allows wearable devices to be worn while on shift
- Willing to wear a smartwatch while on shift
- Have 4 weeks left of study participation or is willing to extend full study participation post 4 months

Procedure:

- Consenting participants were shipped a Garmin smartwatch and were sent instructions on its use via email. Participants were instructed to wear the device while on shift for 4 consecutive weeks. Participants will be able to keep these devices.
- The Garmin (Vivoactive 4) (https://buy.garmin.com/en-CA/CA/p/643382#overview) is a lightweight, smartwatch comprising a slate stainless steel bezel and a silicone band that collects the following metrics: Respiration rate (daytime/night time), SpO2 (daytime/nighttime), HRV stress score (daytime), HR (daytime/nighttime), sleep stages and quality metrics, Duration, intensity, and timing of activities, Inactivity, sedentary time

Supplemental Material: An alternative to the ‘light touch’ digital health remote study: The Stress and Recovery in Frontline COVID-19 Healthcare Workers Study

Hair Cortisol sub-arm

Purpose: To collect hair as a biological measure of chronic stress. Hair grows on average 1 centimeter a month, reflecting approximately 1 month per centimeter of total HPA axis output. Measuring cortisol levels in hair is a preferred option as this collection is pain free, and much less intrusive than other specimen collection for cortisol measurement such as through blood, saliva or urine. Having a retrospective measure of chronic stress enables an additional biological measure of stress to validate our digital biomarkers of stress.

Inclusion criteria:

- Willing to extract 50-60 hairs from the back of the head
- Hair at least 6 centimeters long
- Willing to provide hair extraction on at least 1 occasion, immediately after you consent and at the end of your study participation or later

Exclusion criteria

- Pregnant
- Currently taking glucocorticoid containing medications (e.g., beclomethasone, betamethasone, budesonide, cortisone, dexamethasone, hydrocortisone, methylprednisolone, prednisolone, prednisone, triamcinolone)

Procedure: Interested and consenting participants will be sent a hair cortisol collection kit in the mail along with instructions. Participants will be instructed to extract (with the help of someone else) 50-60 strands of hair from the back of the head and given strategies for extraction to reduce the likelihood of noticeable hair loss. However, 50-60 strands of hair is comparable to the amount lost during hair brushing. Participants will be given a labelled piece of paper and tape and instructed to tape the hair (taking care not to cover the root end with tape) to the piece of paper using the labels provided to indicate which end of hair is the root end. Participants will be provided with pre-posted malling materials to send the hair samples back to the research team at room temperature. We will request that participants complete at least 1 hair extraction, immediately upon consenting to this sub-arm, and possibly again at the end of study participation or in those interested after their study participation.
